# Epithelial and non-epithelial *Ptch1* play opposing roles to regulate proliferation and morphogenesis of the mouse mammary gland

**DOI:** 10.1242/dev.140434

**Published:** 2017-04-01

**Authors:** Teresa Monkkonen, John D. Landua, Adriana P. Visbal, Michael T. Lewis

**Affiliations:** 1 Lester and Sue Smith Breast Center, Baylor College of Medicine, One Baylor Plaza, Houston, TX 77030, USA; 2 Department of Molecular and Cellular Biology, Baylor College of Medicine, One Baylor Plaza, Houston, TX 77030, USA; 3 Program in Developmental Biology, Baylor College of Medicine, One Baylor Plaza, Houston, TX 77030, USA

**Keywords:** Epithelial-stromal interactions, Patched-1, Smoothened, Hedgehog signaling

## Abstract

Patched 1 (*Ptch1*) has epithelial, stromal and systemic roles in murine mammary gland organogenesis, yet specific functions remain undefined. *Cre*-recombinase-mediated *Ptch1* ablation in mammary epithelium increased proliferation and branching, but did not phenocopy transgenic expression of activated smoothened (*SmoM2*). The epithelium showed no evidence of canonical hedgehog signaling, and hyperproliferation was not blocked by smoothened (SMO) inhibition, suggesting a non-canonical function of PTCH1. Consistent with this possibility, nuclear localization of cyclin B1 was increased. In non-epithelial cells, heterozygous *Fsp-Cre-*mediated *Ptch1* ablation increased proliferation and branching, with dysplastic terminal end buds (TEB) and ducts. By contrast, homozygous *Ptch1* ablation decreased proliferation and branching, producing stunted ducts filled with luminal cells showing altered ovarian hormone receptor expression. Whole-gland transplantation into wild-type hosts or estrogen/progesterone treatment rescued outgrowth and hormone receptor expression, but not the histological changes. Bone marrow transplantation failed to rescue outgrowth. Ducts of *Fsp-Cre;Ptch1^fl/fl^* mice were similar to *Fsp-Cre;SmoM2* ducts, but *Fsp-Cre;SmoM2* outgrowths were not stunted, suggesting that the histology might be mediated by *Smo* in the local stroma, with systemic *Ptch1* required for ductal outgrowth and proper hormone receptor expression in the mammary epithelium.

## Abstract (precis)

**Summary:** Systemic and tissue-specific depletion of patched 1 in epithelial and stromal compartments of the mammary gland defines functions in ductal patterning, proliferation and gene expression.

## INTRODUCTION

Organogenesis is the developmental process by which organs are constructed from undifferentiated germ layers. This process requires coordinated interactions between cells and tissues, and, for endocrine-targeted organs, cellular responses to extrinsic hormonal signals. These developmental processes are studied extensively, as they are often perturbed in cancer and other diseases.

The hedgehog signaling network regulates cellular and tissue interactions that are essential for metazoan organogenesis (Briscoe and Thérond, 2013; Johnson et al., 2011; Robbins et al., 2012). In ‘canonical’ mammalian hedgehog signaling, patched 1 (PTCH1) and patched 2 (PTCH2) inhibit downstream signaling by smoothened (SMO), an effector protein, in the absence of ligands. When SMO is inhibited, GLI3, and to a lesser extent GLI2, transcription factors are proteolytically cleaved into transcriptional repressors. With hedgehog ligand [sonic (SHH), indian (IHH) or desert (DHH) hedgehog] binding to PTCH1 and/or PTCH2 (PTCH1/2) on a responding cell, PTCH1/2-mediated inhibition of SMO is released, and GLI transcription factors (GLI1, GLI2 and GLI3) remain full-length transcriptional activators. GLI-mediated transcription regulates proliferation, survival, cell fate and autoregulatory feedback.

Some hedgehog network members function ‘non-canonically’, independent of the signaling cascade described above. For example, PTCH1 can sequester hedgehog ligand to restrict the range of signaling, sequester cyclin B1 in the cytoplasm to inhibit cell cycle progression, or induce caspase 9- or caspase 3-mediated apoptosis in the absence of hedgehog ligands (Barnes et al., 2001; Chen and Struhl, 1996; Mille et al., 2009). In mammary epithelial cells, SHH-stimulated PTCH1 promotes ERK1 and ERK2 phosphorylation independently of SMO (Chang et al., 2010). In the mouse mammary epithelium, constitutively activated *Smo* (*SmoM2*) acts as a G-protein-coupled receptor (GPCR) via G_αi2_ to induce proliferation independently of GLI activity, as hyperproliferation was not blocked by pharmacological inhibition of GLI1 or GLI2 (Villanueva et al., 2015), consistent with observations by Riobo et al. (Riobo et al., 2006). TGFβ induces *Gli2* to regulate osteolysis independently of Smo (Johnson et al., 2011), whereas K-Ras inhibits GLI2 function and GLI3 processing in the context of Smo activation (Lauth et al., 2010). A long non-coding RNA induced by the Twist transcription factor upregulates *Gli1* and *Gas1* (canonical hedgehog target genes) *in vitro* (Zhou et al., 2015). These non-canonical functions necessitate the evaluation of multiple network genes to fully understand hedgehog network function in a given organ.

The murine mammary gland is an excellent model for organogenesis (Daniel and Smith, 1999). In this system, organogenesis is initiated in the embryo, yielding a rudimentary ductal tree at birth, which remains relatively growth quiescent until puberty begins at 3-4 weeks of age. With puberty, systemic hormones (e.g. estrogen, progesterone and other hormones) drive ductal outgrowth via terminal end buds (TEBs). TEBs are transient structures that migrate and proliferate to produce a branched ductal tree that fills the mammary fat pad by 8-10 weeks of age. With conception, pregnancy hormones induce alveolar development to prepare for lactation. After lactation, the gland involutes and remodels to resemble the adult virgin (Hennighausen and Robinson, 2005; Macias and Hinck, 2012).

Previously, analysis of mammary glands from mice heterozygous for a germline knockout allele (*Ptch1^Δ/+^*), or homozygous for a hypomorphic *Ptch1* allele (*Ptch1^mes^*), demonstrated distinct functions for *Ptch1* in the mammary epithelium, local stroma and systemically (mammary gland extrinsic) during postnatal virgin development (Lewis et al., 1999; Moraes et al., 2009). Neither the specific functions of *Ptch1*, nor the association of these phenotypes with canonical hedgehog signaling was investigated. Here, we employ tissue compartment-specific ablation of *Ptch1*, transplantation and tissue-specific expression of an activated *Smo* allele, to specify epithelial, stromal and systemic *Ptch1* functions in virgin mammary gland development.

## RESULTS

### Ptch1 inhibits proliferation and branching of mammary epithelium

To determine the null phenotype of *Ptch1* in mammary epithelium, *mTmG*-tagged primary mammary epithelial cells homo- or heterozygous for a *Ptch1* conditional ablation allele (*Ptch1^fl^*) were treated with Adenovirus-*Cre* (Ad-*Cre*) and transplanted into the mammary fat pads of *SCID/bg* recipients (wild-type for *Ptch1*). Ad-*Cre*-treated, *Ptch1*^+/+^, *mTmG*+ primary cells were transplanted to contralateral fat pads. This approach increased recombination compared with *MMTV-Cre* (Wagner et al., 2001).

**Fig. 1.**
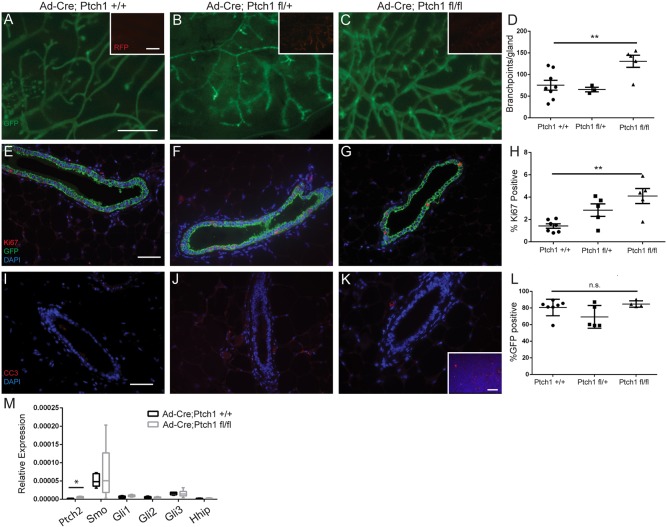
**Loss of *Ptch1* in mammary epithelium increases branching and proliferation in adult virgin glands.** (A-C) Fluorescent whole-mount (A) Ad-*Cre;Ptch1^+/+^*, (B) Ad-*Cre;Ptch1^fl/+^* and (C) Ad-*Cre;Ptch1^fl/fl^* outgrowths. GFP identifies Cre+ cells. The insets show tdTomato Red+ Cre− cells. (D) Quantification showing increased branching in *Ptch1^fl/fl^* epithelium. (E-G) GFP- and Ki67-stained (E) Ad-*Cre;Ptch1^+/+^*, (F) Ad-*Cre;Ptch1^fl/+^* and (G) Ad-*Cre;Ptch1^fl/fl^* ducts. (H) Quantification showing increased proliferation in *Ptch1^fl/fl^* epithelium. (I-K) CC3-stained (I) Ad-*Cre; Ptch1^+/+^*, (J) Ad-*Cre;Ptch1^fl/+^* and (K) Ad-*Cre;Ptch1^fl/fl^* ducts – all negative for CC3. The inset shows a CC3-stained lymph node used as a positive control. (L) Quantification showing similar percentage GFP positivity in outgrowths of different genotypes. (M) Relative expression of hedgehog target genes in Ad-*Cre;Ptch1^+/+^* and *Ptch1^fl/fl^* tissues. Data displayed as 2^−dCt^ with minimum and maximum values. *Ptch2* expression is significantly higher in *Ptch1^fl/fl^* tissues (unpaired *t*-test). Graphs show data as mean±s.e.m. Paired *t*-tests were used to compare *Ptch1^fl/fl^* glands with contralateral *Ptch1^+/+^* controls. *P<0.05 and **P<0.01. Scale bars: 1 mm in A-C; 50 µm in E-G,I-K.

Eight weeks post-transplantation, we observed that whereas *Ptch1^+/+^* glands had 75±11 branch points (Fig. 1A) and *Ptch1^fl/+^* glands had a comparable 65±5 branch points (Fig. 1B), *Ptch1^fl/fl^* glands showed an increase in branch points with 131±14 (Fig. 1C) (*P*<0.011, paired *t*-test; quantification Fig. 1D). Increased branching was present with increased proliferation by Ki67 expression. Eight weeks post-transplantation, 1.4±0.2% of *Ptch1^+/+^* cells were Ki67 positive (Fig. 1E). *Ptch1^fl/+^* ducts were 2.8±0.6% Ki67 positive (Fig. 1F) (n.s., paired *t*-test), whereas proliferation in *Ptch1^fl/fl^* cells increased significantly to 4.1±0.7% Ki67 positive (Fig. 1G) (*P*<0.01, paired *t*-test; quantification, Fig. 1H). Apoptosis was comparable between *Ptch1^+/+^* (Fig. 1I), *Ptch1^fl/+^* (Fig. 1J) or *Ptch1^fl/fl^* ducts (Fig. 1K) using cleaved caspase 3 (CC3) staining, whereas CC3-positive cells were observed in positive control lymph nodes (Fig. 1K, inset).

Previously, the histological defects of *Ptch1^Δ/+^* or *Ptch1^mes^* ducts (Lewis et al., 1999; Moraes et al., 2009) were resolved with epithelial fragment transplantation. Consistently, histology was normal in Ad*-Cre;Ptch1^fl/+^* and Ad*-Cre;Ptch1^fl/fl^* outgrowths (Fig. 1F-G), showing definitively that histological defects were not due to epithelial *Ptch1* loss.

To ensure that the phenotypes were not due to differences in *Cre*-dependent recombination, we determined that GFP-positive cells contributed similarly to ductal outgrowths by immunofluorescence. An average of 81±4% of *Ptch1^+/+^*, 69±6% of *Ptch1^fl/+^* and 85±2% of *Ptch1^fl/fl^* mammary epithelial cells were GFP positive (no difference, paired *t*-test) (Fig. 1L).

**Fig. 2.**
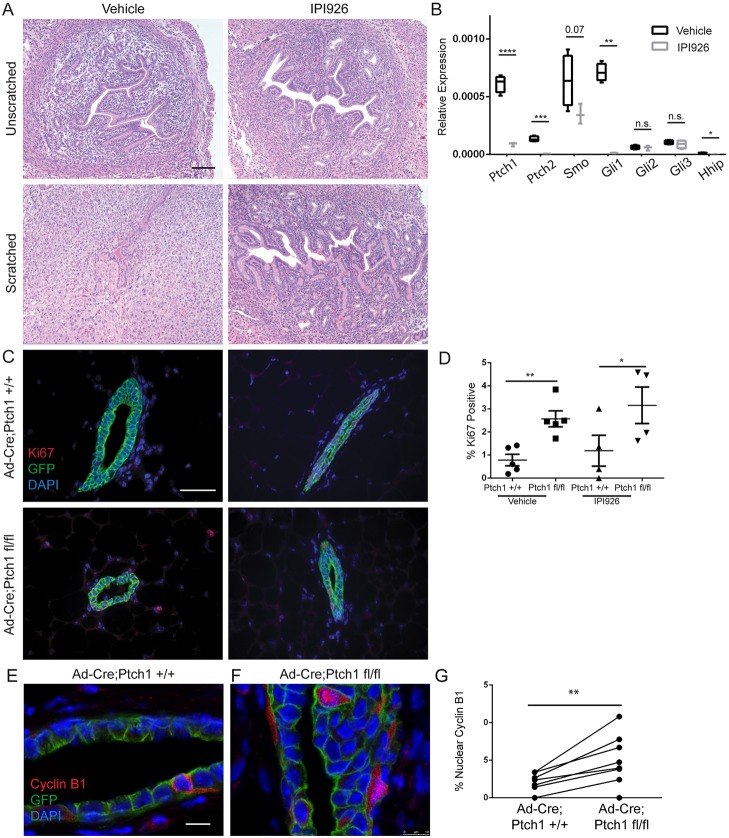
**Hyperproliferation due to Ad-*Cre*-mediated *Ptch1* loss is not due to SMO activation.** (A) Hematoxylin and Eosin-stained vehicle-treated (left panels) and IPI926-treated (right panels) unscratched (upper panels) and scratched (lower panels) uterine tissue, showing decidualization in the vehicle-treated scratched uterus only. (B) qPCR of *Ptch1^+/+^* and *Ptch1^fl/fl^* outgrowths showing no significant changes in hedgehog activation, aside from upregulation of *Ptch2*. Unpaired *t*-test are used for statistics. Data are represented as 2^−dCt^. (C) Ki67 and GFP co-stained vehicle-treated *Ptch1^+/+^* (upper left), vehicle-treated *Ptch1^fl/fl^* (lower left), IPI926-treated *Ptch1^+/+^* (upper right) and IPI926-treated *Ptch1^fl/fl^* ducts (lower right). (D) Quantification of the percentage of Ki67-positive cells by genotype, showing increased proliferation in vehicle and IPI926- treated mutant ducts relative to controls by paired *t*-test. (E,F) Confocal images of cyclin B1- stained *Ptch1^+/+^* and *Ptch1^fl/fl^* ducts. (G) There is increased nuclear localization in *Ptch1^fl/fl^* ducts compared with controls (paired *t*-test). The scatterplot shows data as mean±s.e.m. Boxplots show data as mean±s.e.m., with minimum and maximum values. *P<0.05, **P<0.01, ***P<0.001 and ****P<0.0001. Scale bars: 50 µm in A,C; 10 µm in E,F.

To investigate whether *Ptch1^fl/fl^* outgrowths displayed activated canonical hedgehog signaling due to reduced *Smo* inhibition, *Ptch1^+/+^* and *Ptch1^fl/fl^* epithelium was evaluated by qPCR for hedgehog network gene expression. Of the genes evaluated, only *Ptch2* mRNA was slightly upregulated (Fig. 2F) (*P*<0.016), suggesting that canonical hedgehog signaling was not activated.

### Increased proliferation in Ad-Cre;Ptch1^fl/fl^ ducts is not due to activated canonical hedgehog signaling

Gene expression analysis indicated that phenotypes from *Ptch1* loss may not be due to increased SMO activity (Fig. 1M), consistent with unique mammary gland phenotypes elicited by epithelium-limited ablation of *Ptch1* and activation of *Smo* (Visbal et al., 2011)*.* To test whether hyperproliferation requires Smo activity, we evaluated hyperproliferation due to *Ptch1* loss in the context of pharmacological inhibition of SMO.

To demonstrate SMO inhibitor (IPI926) efficacy, we tested whether IPI926 would blunt uterine scratch-induced decidualization, as canonical hedgehog signaling is required for decidualization (Matsumoto et al., 2002; Villanueva et al., 2015). The unscratched, vehicle- and IPI926-treated uteri displayed comparable histology (Fig. 2A). The scratched vehicle-treated tissue displayed histological changes consistent with decidualization (Fig. 2A) that were absent with IPI926 treatment (Fig. 2A). QPCR supported IPI926 efficacy: *Ptch1*, *Ptch2*, *Gli1* and *Hhip* mRNA levels were significantly reduced in the IPI926-treated, scratched tissue relative to the vehicle-treated, scratched tissue (Fig. 2B).

Given the efficacy of IPI926 *in vivo* at the chosen dose, we treated mice bearing Ad*-Cre;Ptch1^+/+^* and *Ptch1^fl/fl^* outgrowths with IPI926 3 days before harvest at 8 weeks. We assayed for Ki67 to determine whether hyperproliferation was blocked by SMO inhibition. Vehicle-treated *Ptch1^+/+^* ducts were 0.8±0.25% Ki67 positive, and *Ptch1^fl/fl^* ducts increased to 2.6±0.35% Ki67 positive (Fig. 2C; *P*<0.0019, paired *t*-test). With IPI926 treatment, *Ptch1^+/+^* ducts were 1.2±0.6% Ki67 positive, whereas IPI926-treated *Ptch1^fl/fl^* ducts retained increased proliferation with 3.2±0.71% Ki67-positive cells (Fig. 2C) (*P*<0.02, paired *t*-test). No significant differences were observed between vehicle and IPI926-treated *Ptch1^+/+^*, or vehicle and IPI926-treated *Ptch1^fl/fl^* outgrowths (quantification, Fig. 2D). As IPI926 did not block hyperproliferation, this phenotype is not likely due to SMO activation.

As the hyperproliferation with Ad-*Cre*-mediated *Ptch1* loss was not blocked by SMO inhibition, we assayed whether the non-canonical function of PTCH1 in cytoplasmic retention of cyclin B1 could be involved (Barnes et al., 2001). Immunofluorescence showed that whereas *Ptch1^+/+^* ducts displayed 0.17±0.05% cells with nuclear cyclin B1 (Fig. 2E), *Ptch1^fl/fl^* outgrowths showed a significant increase to 0.45±0.11% (Fig. 2F) (*P*<0.0085, paired *t*-test) (Fig. 2G).

### Fsp-cre-mediated disruption of Ptch1 in non-epithelial cells alters mammary gland histology, proliferation and morphology

**Fig. 3.**
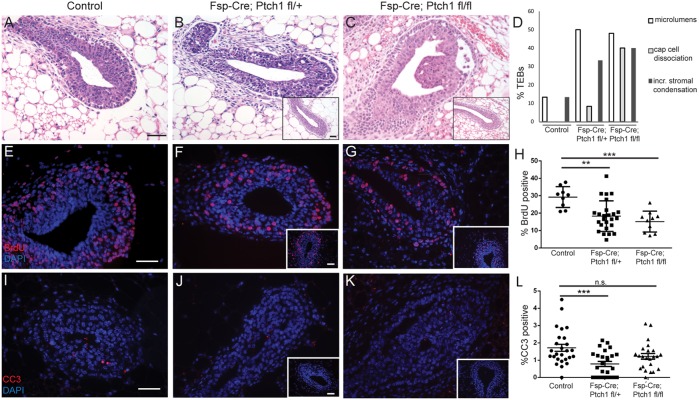
**Pubertal animals (6 weeks) with *Fsp-Cre*-mediated loss of *Ptch1* display dysmorphic hyperproliferative TEBs.** (A-C) Hematoxylin and Eosin-stained (A) control, (B) *Fsp-Cre;Ptch1^fl/+^* and (C) *Fsp-Cre;Ptch1^fl/fl^* TEBs. (D) Percentage perturbed TEBs by genotype. (E-G) BrdU-stained (E) control, (F) *Fsp-Cre;Ptch1^fl/+^* and (G) *Fsp-Cre;Ptch1^fl/fl^* TEBs. (H) Quantification of BrdU in TEBs showing decreased proliferation in both mutants. One data point represents one TEB. (I-K) CC3-stained (I) control, (J) *Fsp-Cre;Ptch1^fl/+^* and (K) *Fsp-Cre;Ptch1^fl/fl^* TEBs. (L) Quantification of CC3 by genotype. Only *Fsp-Cre;Ptch1^fl/+^* has reduced apoptosis. Graphs show data as mean±s.e.m. **P<0.01 and ***P<0.001 by ANOVA/Tukey's test. n.s., not significant. Scale bars: 50 µm. Insets show histologically normal TEBs.

To investigate non-epithelial functions of *Ptch1*, we crossed *mTmG*- tagged, *Ptch1^fl/+^* and *Ptch1^fl/fl^* with *Fsp-Cre* mice to ablate *Ptch1* in fibroblasts and myeloid cells. At 6 weeks of age, control mice (*Fsp-Cre;Ptch1^+/+^* and *Ptch1^fl/+^* or *Ptch1^fl/fl^* mice lacking *Fsp-Cre*) displayed histologically normal TEBs (Fig. 3A), whereas many *Fsp-Cre;Ptch1^fl/+^* TEBs had irregular shape, microlumens and an ill-defined cap cell layer (Fig. 3B). Histologically normal TEBs were also observed (Fig. 3B, inset). Additionally, *Fsp-Cre;Ptch1^fl/fl^* mice showed body cells detached from the cap cells (Fig. 3C), with few histologically normal TEBs (Fig. 3C, inset). Both mutants showed increased stromal condensation adjacent to TEBs relative to controls (Fig. 3B-D).

To test whether the dysmorphic TEBs had altered proliferation, we assayed BrdU labeling. Control TEBs were 29±2% BrdU positive (Fig. 3E), *Fsp-Cre;Ptch1^fl/+^* TEBs were 18±2% positive (Fig. 3F) and *Fsp-Cre;Ptch1^fl/fl^* were 15±2% positive (Fig. 3G). Both mutants had less BrdU labeling than controls (controls versus *Fsp-Cre;Ptch1^fl/+^ P*<0.01, controls versus *Fsp-Cre;Ptch1^fl/fl^ P*<0.001; ANOVA/Tukey's test, Fig. 3H).

With respect to apoptosis, control TEBs were 1.9±0.3% CC3 positive (Fig. 3I), whereas *Fsp-Cre;Ptch1^fl/+^* mice had reduced (0.79±0.14%) CC3 positivity (Fig. 3J) (*P*<0.001, ANOVA/ Tukey's test). *Fsp-Cre;Ptch1^fl/fl^* TEBs had comparable apoptosis rates of 1.5±0.4% (Fig. 3K,L).

**Fig. 4.**
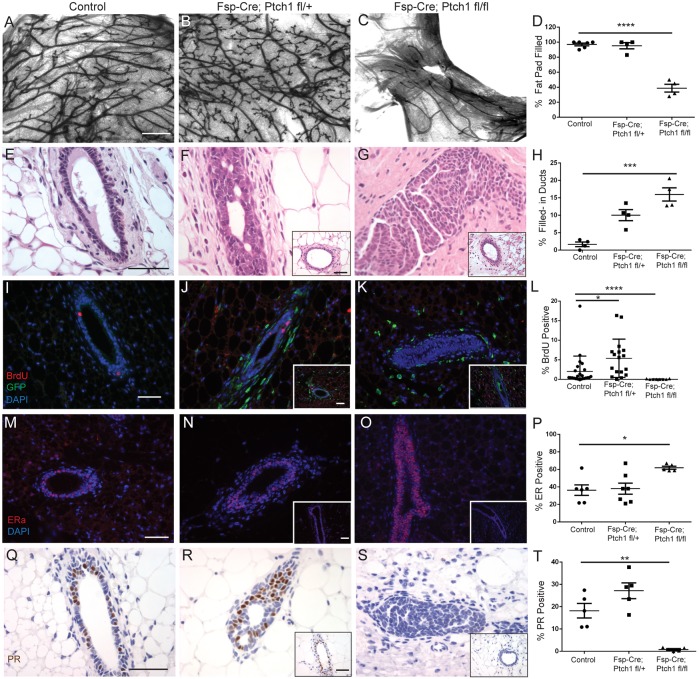
**Eight-week-old control, *Fsp-Cre;Ptch1^fl/+^* and *Fsp-Cre;Ptch1^fl/fl^* animals display altered branching, histology and epithelial proliferation.** (A-C) Whole-mount (A) control, (B) *Fsp-Cre;Ptch1^fl/+^* and (C) *Fsp-Cre;Ptch1^fl/fl^* glands showing branching. Heterozygotes are hyperbranched, whereas homozygotes display reduced branching. (D) Quantification of fat pad filling, showing that *Fsp-Cre;Ptch1^fl/fl^* outgrowths are severely stunted. (E-G) Hematoxylin and Eosin-stained (E) control, (F) *Fsp-Cre;Ptch1^fl/+^* and (G) *Fsp-Cre;Ptch1^fl/fl^* ducts showing frequent partial filling in heterozygotes, and complete filling in homozygotes. (H) Quantification of ductal filling frequency by genotype. (I-K) BrdU-stained (I) control, (J) *Fsp-Cre;Ptch1^fl/+^* and (K) *Fsp-Cre;Ptch1^fl/fl^* ducts. (L) Quantification of BrdU showing hyperproliferation in heterozygotes and hypoproliferation in homozygotes. (M-O) ERα-stained (M) control, (N) *Fsp-Cre;Ptch1^fl/+^* and (O) *Fsp-Cre;Ptch1^fl/fl^* ducts showing upregulated ERα expression in *Fsp-Cre;Ptch1^fl/fl^* ducts. (P) Quantification of ERα in the mammary epithelium. (Q-S) PR-stained (Q) control (R) *Fsp-Cre;Ptch1^fl/+^* and (S) *Fsp-Cre;Ptch1^fl/fl^* ducts showing ablation of PR in *Fsp-Cre;Ptch1^fl/fl^* ducts. (T) Quantification of PR in the mammary epithelium. Graphs show data as mean±s.e.m. Insets display histologically normal ducts. *P<0.05, **P<0.01, ***P<0.001 and ****P<0.0001 by ANOVA/Tukey's test. Scale bars: 1 mm in A-C; 50 µm in E-G,I-K,M-O,Q-S.

In 8-week-old mice, control glands displayed normal branching (66±8 per 2× field) (Fig. 4A). Despite reduced TEB proliferation, *Fsp-Cre;Ptch1^fl/+^* glands were hyperbranched (129±11) (Fig. 4B). By contrast, *Fsp-Cre;Ptch1^fl/fl^* mice had reduced branching (18±4) (Fig. 4C). (Fig. 4A-C show part of the fat pad; quantification in Fig. S1). Control and *Fsp-Cre;Ptch1^fl/+^* fat pads were 95±3% and 95±4% filled with epithelium, respectively, whereas *Fsp-Cre;Ptch1^fl/fl^* ducts were dramatically stunted with 39±5% fat pad filled (*P*<0.0001, ANOVA/Tukey's test) (Fig. 4D). Time points after 8 weeks were not evaluated owing to skin phenotypes and low mutant survival.

Control ducts had normal histology at 8 weeks (Fig. 4E). However, *Fsp-Cre;Ptch1^fl/+^* ducts displayed microlumens and partially filled ducts (Fig. 4F). Histologically normal ducts were also observed (Fig. 4F, inset). *Fsp-Cre;Ptch1^fl/fl^* ducts were more frequently filled (Fig. 4G), with less frequent normal histology (Fig. 4G, inset). There was increased ductal filling in *Fsp-Cre;Ptch1^fl/+^* (*P*<0.01) or *Fsp-Cre;Ptch1^fl/fl^* ducts (*P*<0.001, ANOVA/ Tukey's test) (Fig. 4H).

Ductal filling was confirmed by confocal microscopy of control (Fig. S2A), *Fsp-Cre;Ptch1^fl/+^* (Fig. S2B) and *Fsp-Cre;Ptch1^fl/fl^* glands (Fig. S2C) (Movies 1 and 2 for control and *Fsp-Cre;Ptch1^fl/fl^* ducts). To determine which cell type filled the ducts, we performed immunostaining for cytokeratin 8 (K8) (luminal cells) and cytokeratin 5 (K5) (basal cells). Control ducts had K8+ cells surrounded by K5+ cells as expected (Fig. S2D). *Fsp-Cre;Ptch1^fl/+^* (Fig. S2E) and *Fsp-Cre;Ptch1^fl/fl^* ducts (Fig. S2F) displayed K8+ cells filling ducts. Insets show histologically normal ducts. ZO-1 (zona occludens 1) expression, which stains tight junctions and apical surfaces of luminal cells, was also assayed by immunofluorescence. Although ZO-1 stained the control duct lumens as expected (Fig. S2G), stained *Fsp-Cre;Ptch1^fl/+^* ducts confirmed the presence of microlumens (Fig. S2H), whereas *Fsp-Cre;Ptch1^fl/fl^* ducts displayed abnormal concentric patterning (Fig. S2I).

We observed a significant reduction in mammary gland mass at 8 weeks of age in homozygous mutants (0.04±0.01 g) versus controls (0.16±0.02 g) or heterozygotes (0.13±0.03 g) (Fig. S3A) (*P*<0.01, ANOVA/Tukey's test). Mammary glands of homozygous mutants were also smaller than controls when normalized to body weight (Fig. S3B) (*P*<0.05). *Fsp-Cre;Ptch1^fl/fl^* body weights (14±0.5 g) were also decreased versus controls (23±1.2 g) and heterozygotes (22±0.7 g) (*P*<0.0001, Fig. S3C). Heterozygotes displayed no significant changes.

With respect to proliferation at 8 weeks, control ducts were 2.0±0.8% BrdU positive (Fig. 4I), whereas *Fsp-Cre;Ptch1^fl/+^* ducts were hyperproliferative (5.4±1.2%) (*P*<0.0334, ANOVA/Tukey's test) (Fig. 4J). By contrast, ducts of *Fsp-Cre;Ptch1^fl/fl^* mice showed virtually no proliferation (0.04±0.03%) (Fig. 4K) (*P*<0.0001, ANOVA/Tukey's test; quantification Fig. 4L). Thus, *Fsp-Cre;Ptch1^fl/+^* mammary ducts had increased proliferation and branching, whereas the stunted ducts of *Fsp-Cre;Ptch1^fl/fl^* animals lacked proliferation.

Given that *Fsp-Cre* induces recombination in mammary gland extrinsic cells, and that the stunted duct and hypoproliferation phenotypes observed in *Fsp-Cre;Ptch1^fl/fl^* mice were similar to the stunted hypoproliferative ducts of estrogen receptor α (ERα) knockout mice, and reduced side branching and proliferation similar to the progesterone receptor (PR) knockout mice, we hypothesized that hormone signaling in *Fsp-Cre;Ptch1^fl/fl^* mice was disrupted (Bocchinfuso and Korach, 1997; Lydon et al., 1995).

At 8 weeks of age, ER and PR expression was perturbed in *Fsp-Cre;Ptch1^fl/fl^* mice. Although controls had 36±6% ERα-positive cells (Fig. 4M) and heterozygotes had comparable levels (38±6%) (Fig. 4N), ERα expression in *Fsp-Cre;Ptch1^fl/fl^* ducts increased to 62±2% (Fig. 4O) (*P*<0.05, ANOVA/Tukey's test; quantification, Fig. 4P). Control ducts were 18±3% PR positive (Fig. 4Q) and heterozygotes were comparable (27±4%) (Fig. 4R). However, PR expression was abolished in homozygotes (0.9±0.3%) (Fig. 4S) (*P*<0.01, ANOVA/Tukey's test; quantified in Fig. 4T).

### Whole-gland transplantation rescues ductal growth and ER/PR expression, but not histological defects of Fsp-Cre;Ptch1^fl/fl^ mice

**Fig. 5.**
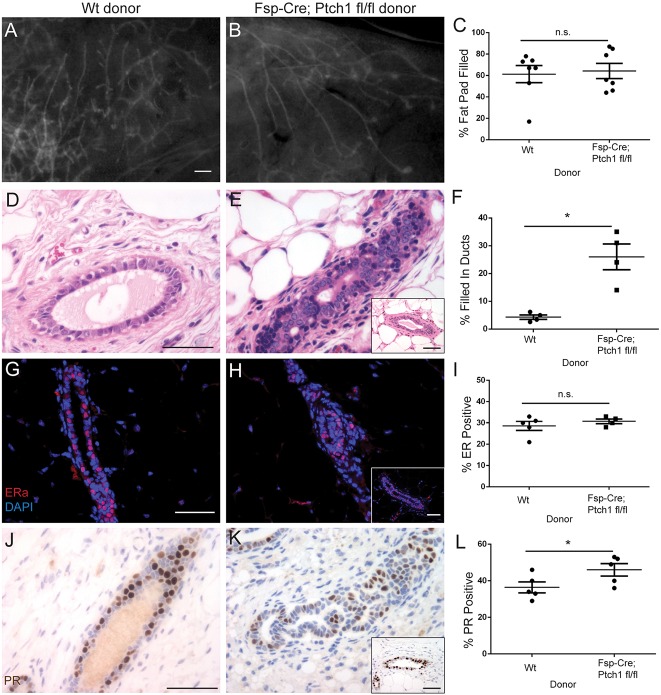
**Whole-gland transplantation rescues stunted ducts and ER/PR levels, but not histology, of *Fsp-Cre;Ptch1^fl/fl^* animals.** Genotype indicates donor glands transplanted to *SCID/bg* recipients that are wild type for *Ptch1*. (A,B) Fluorescent whole-mount (A) control and (B) *Fsp-Cre;Ptch1^fl/fl^* donor glands, 8 weeks post-transplantation. (C) Quantification of fat pad filling, indicating no difference between groups. (D,E) Hematoxylin and Eosin-stained (D) control and (E) *Fsp-Cre;Ptch1^fl/fl^* donor ducts. (F) Quantification of ductal filling, showing increased ductal filling in mutant donors. (G,H) ERα-stained (G) control and (H) *Fsp-Cre;Ptch1^fl/fl^* donor ducts. (I) ERα quantification showing similar expression between groups. (J,K) PR-stained (G) control and (H) *Fsp-Cre;Ptch1^fl/fl^* donor ducts. (L) PR quantification showing a small increase in *Fsp-Cre;Ptch1^fl/fl^* donor ducts. Graphs show data as mean±s.e.m. Insets display histologically normal ducts. *P<0.05 by paired *t*-test. Insets display histologically normal ducts (E,H,K). n.s., not significant. Scale bars: 0.5 mm in A,B; 50 µm in D,E,G,H,J,K.

To determine whether phenotypes caused by *Fsp-Cre*-mediated disruption of *Ptch1* were due to *Ptch1* functions in mammary gland extrinsic cells, *Cre-* control and *Fsp-Cre;Ptch1^fl/fl^* donor glands were transplanted contralaterally into pre-pubertal recipient *SCID/bg* animals that were wild type for *Ptch1*. Eight weeks post-transplantation, the stunted duct phenotype was rescued, with similar fat pad filling between *Cre-* (61±11%) (Fig. 5A) and *Fsp-Cre;Ptch1^fl/fl^* donor glands (64±7%) (Fig. 5B) (quantification, Fig. 5C; *P*<0.8285, paired *t*-test). In contrast to 8-week-old homozygous mutants from genetic crosses (Fig. 4C), TEBs were observed in transplanted *Fsp-Cre;Ptch1^fl/fl^* glands (Fig. 5B). Although *Cre-* donor ducts displayed normal histology (Fig. 5D), *Fsp-Cre;Ptch1^fl/fl^* glands were frequently filled-in (Fig. 5E), with some histologically normal ducts (Fig. 5E inset). *Cre-* donor ducts were 4±0.8% filled, whereas ducts of *Fsp-Cre;Ptch1^fl/fl^* donors were 26±5% filled (*P*<0.0252, paired *t*-test) (quantification, Fig. 5F). Thus, the filled-in duct phenotype is due to loss of *Ptch1* in the local mammary stroma, whereas the stunted duct growth was due to *Ptch1* disruption in mammary gland extrinsic *Fsp*-positive cells.

In whole-gland transplants, ERα positivity was comparable between *Cre-* (29±2%) (Fig. 5G) and *Fsp-Cre;Ptch1^fl/fl^* donor ducts by immunostaining (32±1%) (Fig. 5H) (n.s., paired *t*-test; quantification, Fig. 5I). Similarly, ducts of *Cre-* donors were 36±3% PR positive (Fig. 5J), whereas *Fsp-Cre;Ptch1^fl/fl^* donors were 46±3% (Fig. 5K) (quantification, Fig. 5L). This modest increase was significant (*P*<0.023, paired *t*-test). The normalization of ER and PR levels by whole-gland transplantation demonstrates that mammary gland extrinsic *Ptch1* regulates ductal outgrowth and the characteristic ER/PR patterning of the mammary epithelium.

### Stunted ducts, but not histological defects, of *Fsp-Cre;Ptch1^fl/fl^* mutants are rescued by E+P treatment

**Fig. 6.**
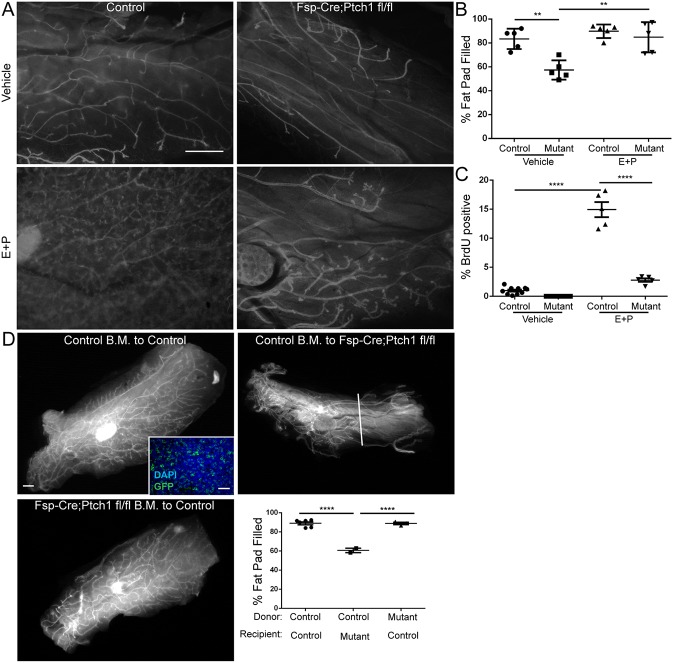
**Ptch1 may regulate estrogen/progesterone production, but not myeloid cell function, to promote mammary ductal elongation.** (A) Whole-mount vehicle- or E+P-treated control or *Fsp-Cre;Ptch1^fl/fl^* glands. E+P increases branching (compare top and bottom panels). (B) Quantification showing E+P-mediated rescue of stunted ducts of *Fsp-Cre;Ptch1^fl/fl^* mutants (‘Mutant’). (C) BrdU labeling quantification in vehicle- or E+P-treated control and *Fsp-Cre;Ptch1^fl/fl^* ducts. E+P induced proliferation, albeit attenuated, in *Fsp-Cre;Ptch1^fl/fl^* mutants. (D) Whole-mount glands of control to control (upper left), control to *Fsp-Cre;Ptch1^fl/fl^* (upper right) or *Fsp-Cre;Ptch1^fl/fl^* to control (lower left) bone marrow-transplanted animals. Inset: inguinal mammary lymph node of Cre- recipient showing colonization by Cre+, mTmG+ cells. Lower right: quantification showing that donor bone marrow does not change mammary ductal outgrowth. Graphs show data as mean±s.e.m. Scale bars: 0.5 mm in A,D; 50 µm in D, inset. **P<0.01, ****P<0.0001 by ANOVA/Tukey's test.

As whole-gland transplantation showed that ‘systemic’ *Ptch1* regulates mammary ductal elongation (Fig. 5) and *Fsp-Cre;Ptch1^fl/fl^* mutants had altered ER/PR patterning (Fig. 4), we tested whether estrogen and progesterone (E+P) treatment would rescue the stunted ducts. Relative to the control vehicle-treated glands, the E+P-treated control glands had increased tertiary branching (Fig. 6A), as expected. Here, *Fsp-Cre;Ptch1^+/+^* and *Ptch1^fl/+^* or *Ptch1^fl/fl^* mice lacking *Fsp-Cre* were used as controls. Although the vehicle-treated control fat pads were 86±4% filled, vehicle-treated mutants displayed reduced fat pad filling (57±4%) and side branching, as previously described (Fig. 6A) (*P*<0.01, ANOVA/Tukey's versus control). E+P-treated control fat pads were 90±3% filled and E+P-treated *Fsp-Cre;Ptch1^fl/fl^* fat pads were 85±6% filled, consistent with rescue of the stunted ducts (quantification, Fig. 6B; *P*<0.01, ANOVA/Tukey's test versus vehicle-treated mutants; no difference, E+P-treated control versus E+P-treated mutants). E+P-treated *Fsp-Cre;Ptch1^fl/fl^* outgrowths still displayed reduced branching compared with the E+P-treated controls (Fig. 6A). Thus, *Ptch1* may regulate estrogen and/or progesterone production to drive pubertal ductal elongation.

We also evaluated proliferation in response to E+P. While vehicle-treated, control glands displayed 1.3±0.3% BrdU positivity, vehicle-treated *Fsp-Cre;Ptch1^fl/fl^* mutants displayed reduced positivity (0±0%) as previously (Fig. 6C). E+P-treated, control ducts were 14.5±1.8% BrdU positive (*P*<0.0001, ANOVA/Tukey's test versus vehicle-treated controls), whereas E+P-treated *Fsp-Cre;Ptch1^fl/fl^* ducts were 2.5±0.5% BrdU positive (Fig. 6C) (significantly reduced versus E+P-treated controls, *P*<0.0001 by ANOVA). Thus, although E+P induced proliferation and branching, the response in *Fsp-Cre;Ptch1^fl/fl^* mutants was attenuated compared with controls. The attenuated proliferation and tertiary branching displayed by the *Fsp-Cre;Ptch1^fl/fl^* mutants in response to E+P suggests that alveologenesis would be perturbed in these animals. Consistent with E+P rescue of ductal outgrowth suggesting functional defects in the ovary, *Fsp-Cre;Ptch1^fl/fl^* animals displayed a disrupted estrous cycle (Fig. S4A-C) and dramatically reduced fertility over 6.5 weeks (Fig. S4D).

### Bone marrow transplantation does not rescue outgrowth of Fsp-Cre;Ptch1^fl/fl^ mutants

Myeloid cells regulate pubertal ductal outgrowth of the mammary epithelium (Gouon-Evans et al., 2000), a subset of which are *Fsp-Cre* positive (Bhowmick et al., 2004). We therefore tested whether bone marrow transplantation could rescue ductal elongation in the *Fsp-Cre;Ptch1^fl/fl^* mutants. Six weeks after transplantation, control recipients of control bone marrow displayed 89±1.5% fat pad filled, and a normal ductal structure (Fig. 6D). Here, controls consisted of *Fsp-Cre;Ptch1^+/+^* and *Ptch1^fl/+^* or *Ptch1^fl/fl^* mice lacking *Fsp-Cre*. We observed engraftment of the transplanted cells, as GFP+ cells were present with transplantation of *Fsp-Cre;Ptch1^+/+^* cells to a *Cre-* recipient (Fig. 6D). Control bone marrow transplanted to *Fsp-Cre;Ptch1^fl/fl^* recipients filled only 61±2% of the fat pad (Fig. 6D). Glands from control recipients of *Fsp-Cre;Ptch1^fl/fl^* bone marrow displayed 89±1.2% of the fat pad filled (Fig. 6D). Control bone marrow transplanted to *Fsp-Cre;Ptch1^fl/fl^* mutants displayed reduced fat pad filling relative to the other groups (Fig. 6D; *P*<0.0001, ANOVA/Tukey's test). The inability of control bone marrow to rescue the mutant phenotype or of mutant bone marrow to induce stunted ducts in controls indicates that *Ptch1* does not regulate ductal elongation in myeloid cells.

### Fsp-Cre-mediated expression of activated SMO phenocopies histological defects in *Fsp-Cre;Ptch1^fl/fl^* mice

**Fig. 7.**
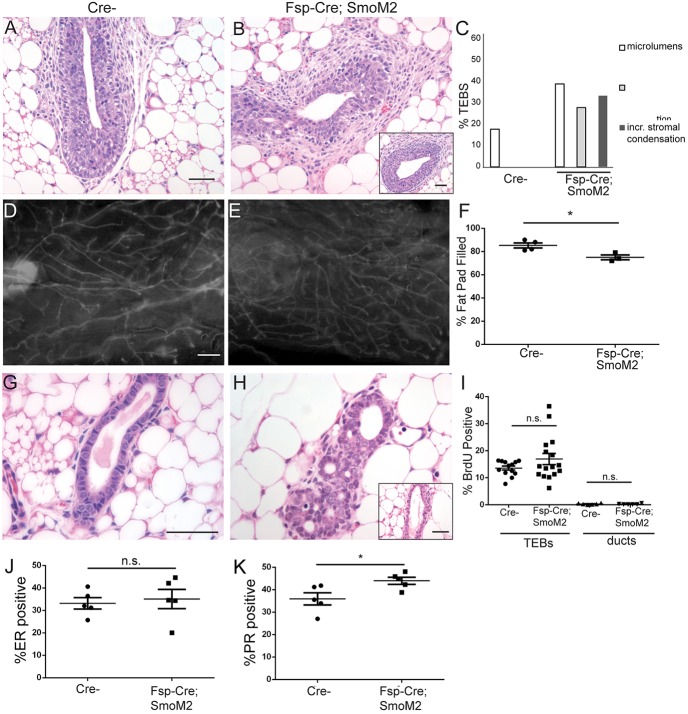
**Aberrant *Fsp-Cre;Ptch1^fl/fl^* histology may be due to activated canonical hedgehog signaling.** (A,B) Hematoxylin and Eosin-stained (A) *Cre-* and (B) *Fsp-Cre; SmoM2* TEBs from 6-week-old mice, showing perturbed histology and increased stromal condensation. (C) Quantification of perturbed TEBs. (D,E) Fluorescent mount of (D) *Cre-* and (E) *Fsp-Cre; SmoM2* glands at 8 weeks. (F) Quantification showing a slight reduction of fat pad filling in mutants. (G,H) Hematoxylin and Eosin-stained (G) *Cre-* and (H) filled-in *Fsp-Cre; SmoM2* ducts. (I) BrdU quantification showing no difference in TEBs at 6 weeks and ducts at 8 weeks. (J) ERα quantification showing no difference at 8 weeks. (K) PR quantification showing a small increase in mutants at 8 weeks. Data are displayed as mean±s.e.m. Unpaired *t*-test was used for analysis. *P<0.05. n.s., not significant. Insets (B,H) show histologically normal structures. Scale bars: 50 µm in A,B,G,H; 0.5 mm in D,E.

To evaluate whether the non-epithelial effects of *Ptch1* loss were possibly mediated by *Smo*, we assessed whether *Fsp-Cre*-mediated expression of activated *Smo* could recapitulate phenotypes in *Fsp-Cre;Ptch1^fl/fl^* mice. At 6 weeks of age, *Cre-* control TEBs had normal histology (Fig. 7A), whereas *Fsp-Cre;SmoM2* TEBs displayed dysmorphia (Fig. 7B). Dysmorphic TEBs had irregular shape, microlumens and increased periductal stromal condensations (Fig. 7C). These TEBs were similar to *Fsp-Cre;Ptch1^fl/+^* (Fig. 3B), *Fsp-Cre;Ptch1^fl/fl^* (Fig. 3C) and *Ptch1^Δ/+^* TEBs (Lewis et al., 1999).

At 8 weeks of age, whole mounts of *Cre-* (Fig. 7D) and *Fsp-Cre;SmoM2*+ glands (Fig. 7E) were comparable. *Cre-* fat pads were 85±2% filled and *Fsp-Cre;SmoM2*+ fat pads were slightly less filled(74±2%) (Fig. 7F) (*P*<0.0225, *t*-test). This reduction was less than in *Fsp-Cre;Ptch1^fl/fl^* mutants, which displayed ∼40% filled fat pads at 8 weeks (Fig. 4D). While *Cre-* ducts at 8 weeks displayed normal histology (Fig. 7G), *Fsp-Cre;SmoM2*+ ducts often contained extra cells and microlumens (Fig. 7H), with some ducts appearing normal (Fig. 7H). Neither mutant TEBs nor mature ducts displayed altered proliferation relative to *Cre-* ducts (Fig. 7I).

We tested whether the ERα and PR expression phenotypes of the *Fsp-Cre;Ptch1^fl/fl^* mutants are phenocopied by the *Fsp-Cre;SmoM2* mutants. At 8 weeks of age, *Cre-* ducts were 33.2±2.5% ER positive and *Fsp-Cre;SmoM2* ducts were 35.1±2.3% positive (Fig. 7J) (not different by *t*-test). PR positivity was 35.9±2.7% in *Cre-* ducts, whereas *Fsp-Cre;SmoM2* ducts displayed slightly higher PR positivity (44.0±1.6%) (Fig. 7K, *P*<0.033, unpaired *t*-test). Thus, *Fsp-Cre;SmoM2* mutants do not display the increased ER or reduced PR expression present in the *Fsp-Cre;Ptch1^fl/fl^* mutants.

## DISCUSSION

**Fig. 8.**
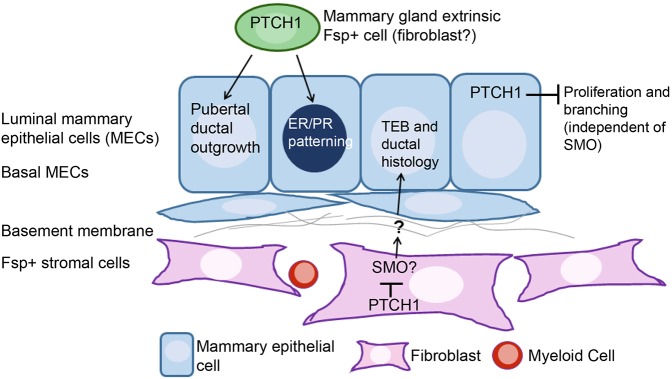
***Ptch1* functions in mammary gland morphogenesis and histogenesis.** PTCH1 in the mammary epithelium inhibits proliferation and branching, independently of SMO. PTCH1 is essential in a mammary gland extrinsic *Fsp*-positive cell (fibroblast) for mammary ductal ER/PR patterning and for pubertal outgrowth. *Ptch1* acts locally in an Fsp-positive stromal cell (likely a fibroblast) to inhibit SMO and elicit normal TEB and ductal histology.

Here, we elucidate tissue compartment-specific roles of *Ptch1* in virgin mammary gland development using improved mouse models, and offer insight into signaling downstream of *Ptch1*. *Ptch1* loss in the mammary epithelium elicits hyperproliferation and hyperbranching, likely independent of Smo. Data from *Fsp-Cre;Ptch1^fl/fl^* mutants indicate *Ptch1* in *Fsp+* fibroblasts regulates ductal histology, perhaps via Smo. We also show the crucial systemic roles of *Ptch1* in ductal elongation and ER/PR expression in the mammary epithelium (see Fig. 8, model).

The Ad-*Cre;Ptch1^fl/fl^* model displayed hyperbranching and hyperproliferation in adult virgins. Whereas mammary glands expressing *SmoM2* also displayed hyperproliferation and hyperbranching (Moraes et al., 2009; Visbal et al., 2011), the *Ptch1* loss and *SmoM2* phenotypes diverge. *SmoM2* expression yielded hyperproliferation and hyperbranching via a mixture of *SmoM2*+ and *SmoM2*– cells (Visbal et al., 2011), and elicited precocious alveolar budding – which are not the case with *Ptch1* loss. Recently, we found that *SmoM2*-dependent hyperproliferation in the mammary gland requires *G_αi2_*-dependent signaling (Villanueva et al., 2015). Hyperproliferation was blocked by inhibiting some G_αi_ subunits, but not by inhibiting GLI1 and GLI2 (Villanueva et al., 2015). The differences between these models suggests that *Ptch1* loss increases proliferation independently of Smo. However, we cannot exclude the possibility that divergent phenotypes could be due to different functions of *SmoM2* [an allele identified in human basal cell carcinoma (Xie et al., 1998)] versus endogenous *Smo*. The phenotypic differences between *SmoM2* conditional expression and *Ptch1* loss in the mammary epithelium agree with the lack of canonical hedgehog target gene upregulation in *Ad-Cre;Ptch1^fl/fl^* ducts, and the inability of IPI926 to block hyperproliferation (Figs 1, 2), suggesting that hyperproliferation is SMO independent. These data fit with reports that SMO (Moraes et al., 2009) and activated hedgehog signaling are absent from the normal mammary epithelium (Chang et al., 2010; Hatsell and Cowin, 2006). From our data, it is possible that *Ptch1* loss-induced hyperproliferation is due to reduced sequestration of cyclin B1 outside the nucleus.

Data here confirm that non-epithelial *Ptch1* regulates ductal histology. Analysis of *Ptch1^Δ/+^* (Lewis et al., 1999) and *Ptch1^mes/mes^* animals (Moraes et al., 2009) indicated that *Ptch1* mediates ductal development; virgin *Ptch1^Δ/+^* mice had dysmorphic TEBs and filled-in ducts (Lewis et al., 1999). Whole *Ptch1^Δ/+^* glands transplanted to a wild-type host displayed filled-in ducts, whereas transplanted epithelial fragments did not, indicating that local stromal *Ptch1* controls histology. From the *Fsp-Cre* model and transplantation experiments, we conclude that *Ptch1* in the mammary fat pad fibroblasts – not myeloid cells – regulates histology. Based on the similar histology of *Fsp-Cre;Ptch1^fl/fl^* and *Fsp-Cre;SmoM2* ducts, it seems that *Ptch1* may regulate histology via Smo. Taken together, the Fsp-Cre and Ad-Cre studies indicate that most phenotypes of the *Ptch1^mes/mes^* mice, including altered TEB and ductal histology, and defective ductal elongation, were due to non-epithelial functions of *Ptch1*.

Aside from defining local stromal *Ptch1* function, we have uncovered a role for mammary extrinsic, non-epithelial Ptch1 in pubertal mammary ductal outgrowth and ER/PR patterning in the mammary epithelium. The *Fsp-Cre;Ptch1^fl/fl^* mutant diverges from the *Ptch1^mes/mes^* mutant (Moraes et al., 2009), which displayed reduced ER and PR expression in stunted ducts. The differences between the *Ptch1^mes/mes^* and *Fsp-Cre;Ptch1^fl/fl^* models could be due to conditional ablation versus a hypomorphic allele, and/or global genetic manipulation versus loss of *Ptch1* in *Fsp*-positive cells. Altered ER/PR patterning may be due to abrogated hormone production by the ovary or pituitary, which may have been differentially affected in these models.

We have also further defined the ‘systemic’ function of *Ptch1*. As E+P rescued the stunted ducts, *Ptch1* may regulate E+P production and ovarian function to regulate pubertal outgrowth and proliferation. Indeed, the *Fsp-Cre;Ptch1^fl/fl^* mutants displayed functional defects, including abrogated cycling and fertility. As the stunted duct phenotype was not rescued by bone marrow transplantation, *Ptch1* does not function in myeloid cells to control ductal elongation.

As *Fsp-Cre*-mediated *Ptch1* loss reduced mammary gland mass, and the mammary fat pad consists primarily of adipocytes, it could be hypothesized that off-target Cre activity in adipocytes contributed to stunted ductal outgrowth. Mice with loss of adipocytes displayed stunted ducts (Landskroner-Eiger et al., 2010). Although we cannot exclude the possibility that changes in the mutant adipocytes contributed to the stunted ducts, we did not observe Cre-dependent GFP expression in adipocytes, consistent with previous reports (Cheng et al., 2005); thus, such effects would likely be due to paracrine signaling.

Data here show stroma-to-epithelium and epithelium intrinsic *Ptch1* functions in mammary gland development. It would be pertinent to determine whether bi-directional hedgehog-mediated tissue interactions exist in other organs where only unidirectional signaling is reported, e.g. prostate and pancreas (Hebrok et al., 2000; Wang et al., 2003). Dissecting these tissue-tissue interactions is crucial, as these developmental programs are inappropriately re-activated in cancer, and correlate with poor prognosis, e.g. in prostate and pancreatic cancer (Bailey et al., 2009; Fan et al., 2004).

### Implications for Ptch1 and Smo in breast cancer

The hedgehog network is misregulated in many cancers, including breast (Moraes et al., 2007; Rubin and de Sauvage, 2006). Although hedgehog network activation induces basal cell carcinoma and medulloblastoma, data connecting hedgehog signaling and breast tumorigenesis are largely correlative, although *Gli1* overexpression in mice induces tumorigenesis (Fiaschi et al., 2009).

PTCH1 protein levels are reduced in 50% of DCIS and invasive breast cancer (IBC), whereas 70% of DCIS and 30% of IBC display aberrant SMO, suggesting that hedgehog activation occurs frequently and early in human breast cancer (Moraes et al., 2007). Furthermore, PTCH1 underexpression correlated with *Ptch1* promoter methylation (Wolf et al., 2007). However, neither *Ptch1^Δ/+^* nor *MMTV-SmoM2* mice show mammary tumors (Moraes et al., 2007,  2009). Our data suggest that perhaps, in the case of *Ptch1^Δ/+^*, the opposing functions of epithelial and systemic *Ptch1* offset one another. These observations may explain why breast cancer incidence in individuals with Gorlin syndrome (Gorlin, 1987), who are heterozygous for germline *Ptch1* loss-of-function and display higher risk for other cancers, is not higher than in the general population. Our Ad-*Cre;Ptch1^fl/+^* data suggest that *Ptch1* heterozygosity would not alter mammary epithelial histology or proliferation.

Previous data suggest that high hedgehog ligand expression in tumor epithelium induces GLI1 (which is indicative of activated hedgehog signaling) in the adjacent stroma, which correlates with invasiveness and poor patient prognosis (O'Toole et al., 2011). As local stromal loss of *Ptch1* and non-epithelial activation of *Smo* promote a DCIS-like phenotype in mammary epithelium, perhaps stromal *Ptch1* loss promotes cancer-associated phenotypes. The data presented here suggest that loss of *Ptch1* in fibroblasts may increase survival, reduce non-apoptotic cell death or alter lumen formation. It would be interesting to determine whether *Ptch1* heterozygosity correlates with DCIS in patients.

## MATERIALS AND METHODS

### Animal models

Mice carrying *Ptch1^c^*, here termed *Ptch1^fl^*, *Cre*-dependent conditional ablation allele were a gift from Dr Brandon Wainwright (University of Queensland, Australia) (Ellis et al., 2003). Mice expressing *Cre*-recombinase under the *Fsp1* (S100A4) promoter were a gift from Dr Eric Neilson (Vanderbilt University, Nashville, TN, USA). These mice express *Cre* in fibroblasts and myeloid-derived leukocytes (Bhowmick et al., 2004). Mice carrying the Gt(ROSA)26Sortm1(Smo/YFP)Amc/J *SmoM2* conditional activation allele were obtained from Jackson Labs (#005130) (Jeong et al., 2004). All animals were genetically tagged with the *mTmG Cre*-dependent reporter at the *Rosa26* locus, Gt(ROSA)26Sortm4(ACTB-tdTomato,−EGFP)Luo/J. Cells lacking *Cre*-recombinase express tdTomato Red, whereas cells expressing *Cre*-recombinase display membrane-bound eGFP (Jackson Labs, #007576) (Muzumdar et al., 2007).

For studies of *Ptch1^fl^*, *Fsp-Cre;Ptch1^fl/+^* males were crossed to *Ptch1^fl/+^* or *Ptch1^fl/fl^* females. *Fsp-Cre;SmoM2* mice were obtained by crossing *Fsp-Cre, mTmG*-positive males to *SmoM2*^+/−^ females (Xie et al., 1998). Genotyping for *Ptch1^fl^*, *SmoM2* and *Fsp-Cre* was performed as previously described (Bhowmick et al., 2004; Ellis et al., 2003; Jeong et al., 2004). CB.17/IcrHsd-Prkdc-scid-Lyst-bg (*SCID/beige*) mice (Harlan Laboratories) used for transplantation were from a breeding colony at Baylor College of Medicine. Animals were maintained according to the NIH Guide for the Care and Use of Experimental Animals with approval from Baylor College of Medicine Institutional Animal Care and Use Committee. For some analyses, 5-Bromo-2′-deoxyuridine (BrdU) (Sigma, B5002) in PBS was administered intraperitoneally 2 h prior to harvest at 250 mg/kg.

### Adenoviral transduction and transplantation

For epithelial ablation of *Ptch1*, mammary epithelial cells were harvested from glands 1, 3, 4 and 5 of 8-week-old *Ptch1^+/+^* and *Ptch1^fl/fl^* females with the lymph nodes removed. Glands were minced, digested with collagenase A (Roche Applied Science) and 0.05% trypsin-EDTA, and strained into single cells (Visbal et al., 2011). Cells were infected at MOI 50 with Adenovirus-*Cre* (Ad-*Cre*) from the Vector Development Laboratory Core Facility at Baylor College of Medicine. Cells were recounted, resuspended in 50% PBS/50% Matrigel (BD Biosciences) and 100,000 *Ptch1^+/+^* and *Ptch1^fl/+^* or *Ptch1^fl/fl^* cells were injected contralaterally into epithelium-free ‘cleared’ inguinal fat pads of 3-week-old SCID/beige recipient mice (Deome et al., 1959) using a Hamilton syringe. Outgrowths were harvested 8 weeks later.

### Whole-mount analysis

For fluorescent whole-mount analysis, glands were agitated in 1 ml of 50% PBS/50% glycerol solution at 4°C overnight as described previously (Landua et al., 2009), and imaged using a Leica MZFL16 fluorescence stereomicroscope with a DFC300 FX camera. Branch points were counted manually using Metamorph software. Confocal microscopy was performed with a Leica TCS SP5 microscope. Non-fluorescent whole mounts were analyzed using Neutral Red (Sigma) staining and imaged with a Leica MZ12.5 stereomicroscope with a Lumenera Infinity 1 camera, as described previously (Landua et al., 2009).

### Immunofluorescence

Tissues were fixed in 4% paraformaldehyde in PBS for 3 h at 4°C, embedded in paraffin wax and sectioned at 3 μm. Slides were rehydrated using decreasing concentrations of ethanol. Immunostaining was carried out using antigen retrieval in 0.1 M sodium citrate buffer (pH 6.0) and heating to 120°C in a decloaker (Biocare Medical). Primary antibodies were incubated overnight at 4°C with 8% MOM protein reagent (Vector Labs, BMK2202) and 1.5% goat serum. See Table S1 for antibody information. Micrographs were taken with a Zeiss Leica Axioskop 2 Plus with an AxioCam MRm FX camera. Cells from ten 40× fields, or ∼1000 mammary epithelial cells were quantified per animal using Metamorph software. Each TEB was a data point, with ∼300 cells/TEB.

### Whole-gland transplantation

Control (*Ptch1^fl/fl^* only or *Fsp-Cre* only) and *Fsp-Cre;Ptch1^fl/fl^* donor glands at 3 weeks of age were transplanted contralaterally into 3-week-old *SCID/bg* recipient mice as described previously (Lewis et al., 2001; Moraes et al., 2009). Glands were analyzed 8 weeks after transplantation.

### Estrogen and progesterone treatment

Daily subcutaneous treatments of 1 µg β-estradiol (Sigma) and 1 mg (Sigma) progesterone in sesame oil, or sesame oil only, were administered for 14 days prior to animal harvest.

### IPI926 treatment (inhibition of SMO)

Either IPI926 (Infinity) dissolved in 13% ethanol in Tween-20 (Sigma) or vehicle alone were administered by oral gavage. IPI926 doses were 40 mg/kg. For the mammary gland experiment, three daily treatments of vehicle or IPI926 were given prior to harvest.

### Uterine scratch

After ovarectomy post-weaning and a 1-week rest, a prescribed course of estrogen (0.1 µg in 100 µl sesame oil for 3 days), 2 days rest, then estrogen+progesterone (1 mg progesterone+6.7 ng estrogen daily until harvest) was administered prior to scratch of one uterine horn by blunted needle as described previously (Finn and Martin, 1972). Vehicle or IPI926 was administered for 7 days prior to, and the day of harvest 9 days after the first estrogen treatment. Hormone and IPI926 doses were timed as described previously (Villanueva et al., 2015).

### QPCR

Tissues were collected into RNA Later (Qiagen) and frozen at −80°C. RNA was extracted with the Qiagen RNeasy Kit, and cDNA was synthesized with the Superscript III kit (Thermo Fisher) using random hexamers. The cDNA was analyzed using an Applied Biosystems 7500-Fast thermocycler for TaqMan quantitative PCR under standard conditions. Product accumulation was represented as 2^−ΔCt^, with ANOVA of ΔCt values used for statistical comparison. 18S rRNA was used for normalization. See Table S2 for primers.

### Bone marrow transplantation

Recipient animals 4-5 weeks of age received Baytril water 24 h prior to irradiation and up to 6 days post-irradiation. Recipients received a dose of 400 centigray, and 24 h later, bone marrow cells were harvested and isolated from 4-5-week-old donor mice. Irradiated recipients received 2 million donor cells injected retro-orbitally. Recipients were harvested 6 weeks post-transplantation.

## References

[DEV140434C1] Bailey J. M., Mohr A. M. and Hollingsworth M. A. (2009). Sonic hedgehog paracrine signaling regulates metastasis and lymphangiogenesis in pancreatic cancer. *Oncogene* 28, 3513-3525. 10.1038/onc.2009.220 19633682 PMC2910592

[DEV140434C2] Barnes E. A., Kong M., Ollendorff V. and Donoghue D. J. (2001). Patched1 interacts with cyclin B1 to regulate cell cycle progression. *EMBO J.* 20, 2214-2223. 10.1093/emboj/20.9.2214 11331587 PMC125436

[DEV140434C3] Bhowmick N. A., Chytil A., Plieth D., Gorska A. E., Dumont N., Shappell S., Washington M. K., Neilson E. G. and Moses H. L. (2004). TGF-beta signaling in fibroblasts modulates the oncogenic potential of adjacent epithelia. *Science* 303, 848-851. 10.1126/science.1090922 14764882

[DEV140434C4] Bocchinfuso W. P. and Korach K. S. (1997). Mammary gland development and tumorigenesis in estrogen receptor knockout mice. *J. Mammary Gland Biol. Neoplasia.* 2, 323-334. 10.1023/A:1026339111278 10935020

[DEV140434C5] Briscoe J. and Thérond P. P. (2013). The mechanisms of Hedgehog signalling and its roles in development and disease. *Nat. Rev. Mol. Cell Biol.* 14, 416-429. 10.1038/nrm3598 23719536

[DEV140434C6] Chang H., Li Q., Moraes R. C., Lewis M. T. and Hamel P. A. (2010). Activation of Erk by sonic hedgehog independent of canonical hedgehog signalling. *Int. J. Biochem. Cell Biol.* 42, 1462-1471. 10.1016/j.biocel.2010.04.016 20451654 PMC3038129

[DEV140434C7] Chen Y. and Struhl G. (1996). Dual roles for patched in sequestering and transducing Hedgehog. *Cell* 87, 553-563. 10.1016/S0092-8674(00)81374-4 8898207

[DEV140434C8] Cheng N., Bhowmick N. A., Chytil A., Gorksa A. E., Brown K. A., Muraoka R., Arteaga C. L., Neilson E. G., Hayward S. W. and Moses H. L. (2005). Loss of TGF-beta type II receptor in fibroblasts promotes mammary carcinoma growth and invasion through upregulation of TGF-alpha-, MSP- and HGF-mediated signaling networks. *Oncogene* 24, 5053-5068. 10.1038/sj.onc.1208685 15856015 PMC3074577

[DEV140434C9] Daniel C. W. and Smith G. H. (1999). The mammary gland: a model for development. *J. Mammary Gland Biol. Neoplasia.* 4, 3-8. 10.1023/A:1018796301609 10219902

[DEV140434C10] Deome K. B., Faulkin L. J. Jr, Bern H. A. and Blair P. B. (1959). Development of mammary tumors from hyperplastic alveolar nodules transplanted into gland-free mammary fat pads of female C3H mice. *Cancer Res* 19, 515-520. 13663040

[DEV140434C11] Ellis T., Smyth I., Riley E., Graham S., Elliot K., Narang M., Kay G. F., Wicking C. and Wainwright B. (2003). Patched 1 conditional null allele in mice. *Genesis* 36, 158-161. 10.1002/gene.10208 12872247

[DEV140434C12] Fan L., Pepicelli C. V., Dibble C. C., Catbagan W., Zarycki J. L., Laciak R., Gipp J., Shaw A., Lamm M. L. G., Munoz A. et al. (2004). Hedgehog signaling promotes prostate xenograft tumor growth. *Endocrinology* 145, 3961-3970. 10.1210/en.2004-0079 15132968

[DEV140434C13] Fiaschi M., Rozell B., Bergström A. and Toftgård R. (2009). Development of mammary tumors by conditional expression of GLI1. *Cancer Res.* 69, 4810-4817. 10.1158/0008-5472.CAN-08-3938 19458072 PMC2689922

[DEV140434C14] Finn C. A. and Martin L. (1972). Endocrine control of the to timing of endometrial stimulus sensitivity a decidual to implantation importance hormones later. *Biol. Reprod.* 7, 82-86. 10.1093/biolreprod/7.1.82 5050152

[DEV140434C15] Gorlin R. J. (1987). Nevoid basal cell carcinoma syndrome. *Medicine* 66, 98-113. 10.1097/00005792-198703000-00002 3547011

[DEV140434C16] Gouon-Evans V., Rothenberg M. E. and Pollard J. W. (2000). Postnatal mammary gland development requires macrophages and eosinophils. *Development* 127, 2269-2282. 10804170 10.1242/dev.127.11.2269

[DEV140434C17] Hatsell S. J. and Cowin P. (2006). Gli3-mediated repression of Hedgehog targets is required for normal mammary development. *Development* 133, 3661-3670. 10.1242/dev.02542 16914490

[DEV140434C18] Hebrok M., Kim S. K., St Jacques B., McMahon A. P. and Melton D. A. (2000). Regulation of pancreas development by hedgehog signaling. *Development* 127, 4905-4913. 11044404 10.1242/dev.127.22.4905

[DEV140434C19] Hennighausen L. and Robinson G. W. (2005). Information networks in the mammary gland. *Nat. Rev. Mol. Cell Biol.* 6, 715-725. 10.1038/nrm1714 16231422

[DEV140434C20] Jeong J., Mao J., Tenzen T., Kottmann A. H. and McMahon A. P. (2004). Hedgehog signaling in the neural crest cells regulates the patterning and growth of facial primordia. *Genes Dev.* 18, 937-951. 10.1101/gad.1190304 15107405 PMC395852

[DEV140434C21] Johnson R. W., Nguyen M. P., Padalecki S. S., Grubbs B. G., Merkel A. R., Oyajobi B. O., Matrisian L. M., Mundy G. R. and Sterling J. A. (2011). TGF-beta promotion of Gli2-induced expression of parathyroid hormone-related protein, an important osteolytic factor in bone metastasis, is independent of canonical Hedgehog signaling. *Cancer Res.* 71, 822-831. 10.1158/0008-5472.CAN-10-2993 21189326 PMC3077118

[DEV140434C22] Landskroner-Eiger S., Park J., Israel D., Pollard J. W. and Scherer P. E. (2010). Morphogenesis of the developing mammary gland: stage-dependent impact of adipocytes. *Dev. Biol.* 344, 968-978. 10.1016/j.ydbio.2010.06.019 20599899 PMC2917626

[DEV140434C23] Landua J. D., Visbal A. P. and Lewis M. T. (2009). Methods for preparing fluorescent and neutral red-stained whole mounts of mouse mammary glands. *J. Mammary Gland Biol. Neoplasia.* 14, 411-415. 10.1007/s10911-009-9155-3 19936989 PMC3038127

[DEV140434C24] Lauth M., Bergström Å., Shimokawa T., Tostar U., Jin Q., Fendrich V., Guerra C., Barbacid M. and Toftgård R. (2010). DYRK1B-dependent autocrine-to-paracrine shift of Hedgehog signaling by mutant RAS. *Nat. Struct. Mol. Biol.* 17, 718-725. 10.1038/nsmb.1833 20512148

[DEV140434C25] Lewis M. T., Ross S., Strickland P. A., Sugnet C. W., Jimenez E., Scott M. P. and Daniel C. W. (1999). Defects in mouse mammary gland development caused by conditional haploinsufficiency of Patched-1. *Development* 126, 5181-5193. 10529434 10.1242/dev.126.22.5181

[DEV140434C26] Lewis M. T., Ross S., Strickland P. A., Sugnet C. W., Jimenez E., Hui C.-c. and Daniel C. W. (2001). The Gli2 transcription factor is required for normal mouse mammary gland development. *Dev. Biol.* 238, 133-144. 10.1006/dbio.2001.0410 11783999

[DEV140434C27] Lydon J. P., DeMayo F. J., Funk C. R., Mani S. K., Hughes A. R., Montgomery C. A., Shyamala G., Conneely O. M. and O'Malley B. W. (1995). Mice lacking progesterone receptor exhibit pleiotropic reproductive abnormalities. *Genes Dev.* 9, 2266-2278. 10.1101/gad.9.18.2266 7557380

[DEV140434C28] Macias H. and Hinck L. (2012). Mammary gland development. *Wiley Interdiscip. Rev. Dev. Biol.* 1, 533-557. 10.1002/wdev.35 22844349 PMC3404495

[DEV140434C29] Matsumoto H., Zhao X., Das S. K., Hogan B. L. M. and Dey S. K. (2002). Indian hedgehog as a progesterone-responsive factor mediating epithelial-mesenchymal interactions in the mouse uterus. *Dev. Biol.* 245, 280-290. 10.1006/dbio.2002.0645 11977981

[DEV140434C30] Mille F., Thibert C., Fombonne J., Rama N., Guix C., Hayashi H., Corset V., Reed J. C. and Mehlen P. (2009). The Patched dependence receptor triggers apoptosis through a DRAL-caspase-9 complex. *Nat. Cell Biol.* 11, 739-746. 10.1038/ncb1880 19465923 PMC2844407

[DEV140434C31] Moraes R. C., Zhang X., Harrington N., Fung J. Y., Wu M.-F., Hilsenbeck S. G., Allred D. C. and Lewis M. T. (2007). Constitutive activation of smoothened (SMO) in mammary glands of transgenic mice leads to increased proliferation, altered differentiation and ductal dysplasia. *Development* 134, 1231-1242. 10.1242/dev.02797 17287253

[DEV140434C32] Moraes R. C., Chang H., Harrington N., Landua J. D., Prigge J. T., Lane T. F., Wainwright B. J., Hamel P. A. and Lewis M. T. (2009). Ptch1 is required locally for mammary gland morphogenesis and systemically for ductal elongation. *Development* 136, 1423-1432. 10.1242/dev.023994 19297414 PMC2675781

[DEV140434C33] Muzumdar M. D., Tasic B., Miyamichi K., Li L. and Luo L. (2007). A global double-fluorescent Cre reporter mouse. *Genesis* 605, 593-605. 10.1002/dvg.20335 17868096

[DEV140434C34] O'Toole S. A., Machalek D. A., Shearer R. F., Millar E. K. A., Nair R., Schofield P., McLeod D., Cooper C. L., McNeil C. M., McFarland A. et al. (2011). Hedgehog overexpression is associated with stromal interactions and predicts for poor outcome in breast cancer. *Cancer Res.* 71, 4002-4014. 10.1158/0008-5472.CAN-10-3738 21632555

[DEV140434C35] Riobo N. A., Saucy B., Dilizio C. and Manning D. R. (2006). Activation of heterotrimeric G proteins by Smoothened. *Proc. Natl. Acad. Sci. USA* 103, 12607-12612. 10.1073/pnas.0600880103 16885213 PMC1567926

[DEV140434C36] Robbins D. J., Fei D. L. and Riobo N. A. (2012). The Hedgehog signal transduction network. *Sci. Signal.* 5, re6 10.1126/scisignal.2002906 23074268 PMC3705708

[DEV140434C37] Rubin L. L. and de Sauvage F. J. (2006). Targeting the Hedgehog pathway in cancer. *Nat. Rev. Drug Discov.* 5, 1026-1033. 10.1038/nrd2086 17139287

[DEV140434C38] Villanueva H., Visbal A. P., Obeid N. F., Ta A. Q., Faruki A. A., Wu M.-F., Hilsenbeck S. G., Shaw C. A., Yu P., Plummer N. W. et al. (2015). An essential role for Gα(i2) in Smoothened-stimulated epithelial cell proliferation in the mammary gland. *Sci. Signal.* 8, ra92 10.1126/scisignal.aaa7355 26373672

[DEV140434C39] Visbal A. P., LaMarca H. L., Villanueva H., Toneff M. J., Li Y., Rosen J. M. and Lewis M. T. (2011). Altered differentiation and paracrine stimulation of mammary epithelial cell proliferation by conditionally activated Smoothened. *Dev. Biol.* 352, 116-127. 10.1016/j.ydbio.2011.01.025 21276786 PMC3057274

[DEV140434C40] Wagner K.-U., Ward T., Davis B., Wiseman R. and Hennighausen L. (2001). Spatial and temporal expression of the Cre gene under the control of the MMTV-LTR in different lines of transgenic mice. *Transgenic Res.* 10, 545-553. 10.1023/A:1013063514007 11817542

[DEV140434C41] Wang B. E., Shou J., Ross S., Koeppen H., De Sauvage F. J. and Gao W.-Q. (2003). Inhibition of epithelial ductal branching in the prostate by sonic hedgehog is indirectly mediated by stromal cells. *J. Biol. Chem.* 278, 18506-18513. 10.1074/jbc.M300968200 12626524

[DEV140434C42] Wolf I., Bose S., Desmond J. C., Lin B. T., Williamson E. A., Karlan B. Y. and Koeffler H. P. (2007). Unmasking of epigenetically silenced genes reveals DNA promoter methylation and reduced expression of PTCH in breast cancer. *Breast Cancer Res.* 105, 139-155. 10.1007/s10549-006-9440-4 17295047

[DEV140434C43] Xie J., Murone M., Luoh S.-M., Ryan A., Gu Q., Zhang C., Bonifas J. M., Lam C.-W., Hynes M., Goddard A. et al. (1998). Activating Smoothened mutations in sporadic basal-cell carcinoma. *Nature* 391, 90-92. 10.1038/34201 9422511

[DEV140434C44] Zhou M., Hou Y., Yang G., Zhang H., Tu G., Du Y., Wen S., Xu L., Tang X., Tang S. et al. (2015). LncRNA-Hh strengthen cancer stem cells generation in twist-positive breast cancer via activation of hedgehog signaling pathway. *Stem Cells* 34, 55-66. 10.1002/stem.2219. 26418365 PMC4832137

